# c-Met-Specific Chimeric Antigen Receptor T Cells Demonstrate Anti-Tumor Effect in c-Met Positive Gastric Cancer

**DOI:** 10.3390/cancers13225738

**Published:** 2021-11-16

**Authors:** Chung Hyo Kang, Yeongrin Kim, Da Yeon Lee, Sang Un Choi, Heung Kyoung Lee, Chi Hoon Park

**Affiliations:** 1 Therapeutics & Biotechnology Division, Korea Research Institute of Chemical Technology, Daejeon 34114, Korea; kangch@vigencell.com (C.H.K.); kyr3915@krict.re.kr (Y.K.); ekdus727@krict.re.kr (D.Y.L.); suchoi@krict.re.kr (S.U.C.); craet@krict.re.kr (H.K.L.); 2 Medicinal Chemistry and Pharmacology, Korea University of Science and Technology, Daejeon 34113, Korea; 3 College of Pharmacy, Chungnam National University, Daejeon 34134, Korea

**Keywords:** c-Met, gastric cancer, chimeric antigen receptor, CAR T cell, KHYG-1

## Abstract

**Simple Summary:**

c-Met is known to be overexpressed in gastric cancers. Here, we developed anti-c-Met CAR T cell and measured its anti-tumor efficacy in vitro and in vivo. Our anti c-Met CAR T cells have shown selective killing of c-Met overexpressed gastric cancer cells. Based on our results, we suggest that anti-c-Met CAR T cell therapy could be effective for gastric cancer patients.

**Abstract:**

Chimeric antigen receptor (CAR) technology has been highlighted in recent years as a new therapeutic approach for cancer treatment. Although the impressive efficacy of CAR-based T cell adoptive immunotherapy has been observed in hematologic cancers, limited effect has been reported on solid tumors. Approximately 20% of gastric cancer (GC) patients exhibit a high expression of c-Met. We have generated an anti c-Met CAR construct that is composed of a single-chain variable fragment (scFv) of c-Met antibody and signaling domains consisting of CD28 and CD3ζ. To test the CAR construct, we used two cell lines: the Jurkat and KHYG-1 cell lines. These are convenient cell lines, compared to primary T cells, to culture and to test CAR constructs. We transduced CAR constructs into Jurkat cells by electroporation. c-Met CAR Jurkat cells secreted interleukin-2 (IL-2) only when incubated with c-Met positive GC cells. To confirm the lytic function of CAR, the CAR construct was transduced into KHYG-1, a NK/T cell line, using lentiviral particles. c-Met CAR KHYG-1 showed cytotoxic effect on c-Met positive GC cells, while c-Met negative GC cell lines were not eradicated by c-Met CAR KHYG-1. Based on these data, we created c-Met CAR T cells from primary T cells, which showed high IL-2 and IFN-γ secretion when incubated with the c-Met positive cancer cell line. In an in vivo xenograft assay with NSG bearing MKN-45, a c-Met positive GC cell line, c-Met CAR T cells effectively inhibited the tumor growth of MKN-45. Our results show that the c-Met CAR T cell therapy can be effective on GC.

## 1. Introduction

Research on CAR T cell therapy has become quite active since CAR T cells targeting CD19 have shown an impressive response rate in patients with B-cell acute lymphoblastic leukemia (B-ALL) [1,2,3,4,5,6]. CAR T cell therapy is an adoptive immunotherapy technique that reinjects engineered T cells into a patient. CAR is composed of an scFv domain for antigen recognition, a co-stimulatory domain, and a CD3ζ signaling domain for T cell activation. T cells that stably express the CAR construct are able to specifically recognize the antigen and effectively eliminate the tumor cells [7,8,9,10]. Despite the striking therapeutic effect on hematologic cancers, CAR T cell therapy has little effect on solid cancers due to immunosuppressive tumor microenvironments (TMEs), insufficient tumor trafficking, and a deficiency of cancer specific antigens [11,12].

Gastric cancer (GC) is the fourth most common cancer after lung cancer, breast cancer, and colorectal cancer worldwide, and is a dangerous cancer with a high mortality rate [13]. Older people are at higher risk for cancer progression, which usually occurs between ages 50 and 80. Approximately 20% of GC patients show overexpression of c-Met [14,15,16]. c-Met, also the called hepatocyte growth factor receptor (HGFR), has a high affinity for its ligand, hepatocyte growth factor (HGF) [17]. The c-Met consists of α- and β-subunits, and the sema domain of the β-subunit is responsible for signaling [18]. Overexpression or dysregulation of c-Met is found in various cancer types, including gastric, prostate, colon, lung, and breast cancer [19,20,21,22,23]. Abnormal signaling pathway caused by c-Met overexpression is involved in cell survival, proliferation, migration, and angiogenesis, leading to tumor progression, invasion, metastasis, and recurrence [24]. For this reason, c-Met is regarded as a potent target for cancer treatment, and various small molecules of c-Met inhibitors are being developed [25,26]. However, no selective c-Met inhibitor has shown efficacy in clinical trials [27]. Because c-Met overexpression occurs most often in GC compared to other cancer types, we thought that c-Met CAR T cell therapy against GC could be successful. To prove this, we tested c-Met CAR T cells on various gastric cancer cell lines in this study.

Here, we investigated the CAR T cell therapy for the treatment of c-Met overexpressed GC. In this study, we developed a c-Met CAR construct, which is composed of anti c-Met scFv, CD28, and CD3ζ. Two cell lines, the Jurkat CD4+ T cell line and the KHYG-1 NK/T cell line, were used to test the CAR construct. Our c-Met CAR T cell exhibited an anti-tumor effect against c-Met overexpressing GC in vitro and in vivo.

## 2. Results

### 2.1. Development of the c-Met CAR Constructs

**Figure 1:**
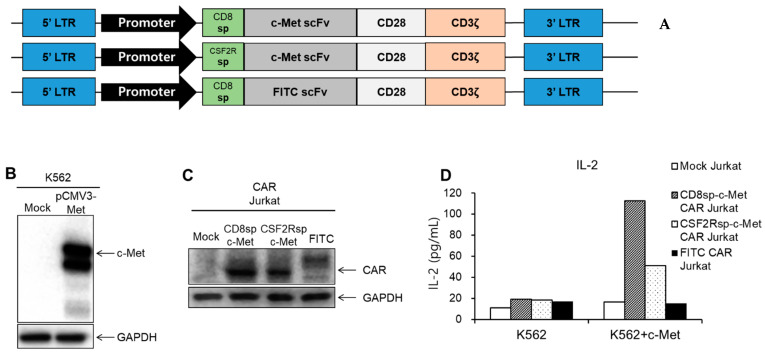
Generation of CARs and analysis of IL-2 secretion by c-Met CAR Jurkat cell. (**A**) Schematic representation of c-Met CAR and FITC CAR construct. (**B**) c-Met gene (pCMV3-Met) was transduced by electroporation into K562, and Western blot was performed. (**C**) After transducing the mock CD8sp-c-Met CAR, CSF2Rsp-c-Met CAR, or FITC CAR construct into Jurkat, expressions of CAR were confirmed through Western blot. (**D**) The mock CD8sp-c-Met CAR, CSF2Rsp-c-Met CAR, or FITC CAR Jurkat were co-cultured with K562 or c-Met-K562 at an E:T ratio of 15:1. After overnight incubation, supernatants were collected and ELISA was performed to measure IL-2 level. (The whole western blots figure see Figure S1).

We developed anti-c-Met CAR constructs composed of an anti-c-Met scFv, which is from patent KR10-1615619, and signaling domains consisting of CD28 and CD3ζ (Figure 1A, Supplementary Material S1). An anti-FITC CAR capable of recognizing fluorescein isothiocyanate (FITC) was generated to be used as a negative control. We created two c-Met CAR constructs, CD8sp-c-Met CAR and CSF2Rsp-c-Met CAR, which have the CD8 signaling peptide and CSF2R signaling peptide, respectively.

To assess whether c-Met CAR specifically recognizes the c-Met antigen, we used the K562-c-Met cell line as a target cell (Figure 1B). CD8sp-c-Met CAR, CSF2Rsp-c-Met CAR, or FITC CAR was transiently transduced into the Jurkat cell (Figure 1C). Jurkat is a T lymphocyte cell line, which secretes IL-2 when activated by antigen recognition. The amount of IL-2 secreted by the Jurkat cell indicates whether the CAR Jurkat cells are activated by recognizing target cells. The CD8sp-c-Met CAR, CSF2Rsp-c-Met CAR, or FITC CAR Jurkat cells were co-cultured with c-Met negative K562 or c-Met positive K562-c-Met cells in 96-well plates, respectively. After overnight incubation, the amount of IL-2 present in the culture medium was measured by enzyme-linked immunosorbent assay (ELISA) (Figure 1D). As a result, both c-Met CAR Jurkat cells secreted high level of IL-2 when incubated with K562-c-Met cells, while little IL-2 was secreted when c-Met CAR Jurkat cells were incubated with K562 cells. Through this, we confirmed that both c-Met CARs are able to recognize c-Met antigen and activate T cells.

### 2.2. c-Met Positive GC Cells Induced Activation of c-Met CAR Jurkat and KHYG-1 Cells

**Figure 2:**
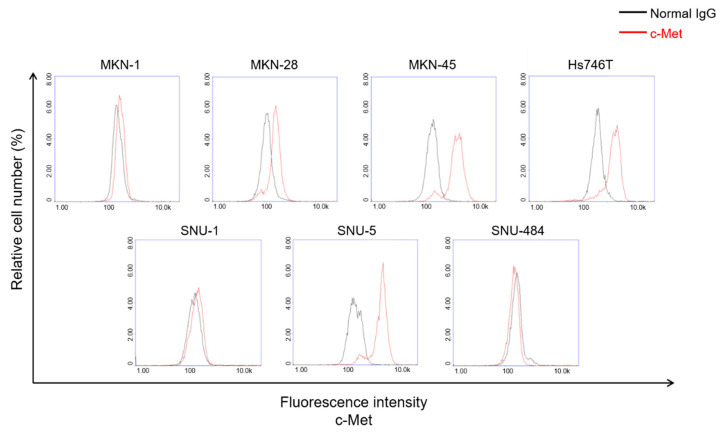
Analysis of surface c-Met expression in several GC cell lines. Several GC cell lines were stained with c-Met antibody to see the c-Met level on the cell surface.

**Figure 3:**
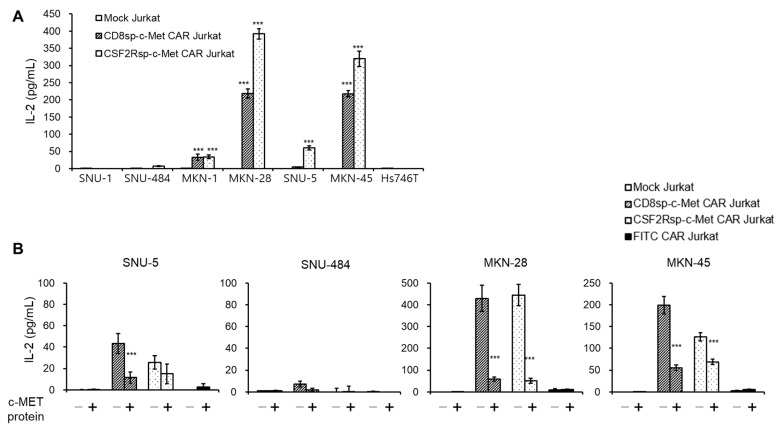
c-Met CAR Jurkat specifically recognizes the c-Met positive GC cells. (**A**) The mock CD8sp-c-Met CAR, or CSF2Rsp-c-Met CAR Jurkat cells (1.5 × 10^5^ cells) were co-cultured with GC cell lines (1 × 10^4^ cells) at an E:T ratio of 15:1. After overnight incubation, supernatants were collected and ELISA was performed to measure IL-2 level. The bars show mean ± S.D. of experiments performed in triplicate. Statistical analysis was performed by the paired *t*-test. *** *p* ≤ 0.01, compared with the mock Jurkat group. (**B**) The mock CD8sp-c-Met CAR, CSF2Rsp-c-Met CAR, or FITC CAR Jurkat cells were co-cultured with GC cell lines in the presence or absence of purified soluble c-MET protein. The next day, supernatants were collected and IL-2 ELISA was performed. The bars show mean ± S.D, of experiments performed in triplicate. Statistical analysis was performed by the paired *t*-test. *** *p* ≤ 0.01, compared with the c-Met absence group.

Next, we analyzed the expression level of c-Met protein on the cell surface in a number of GC cell lines (Figure 2). Based on the c-Met expression data, we classified c-Met positive or c-Met negative cells. Our data indicated that no c-Met was found on the cell in the SNU-1 and SNU484 cell lines, low levels of surface c-Met were found in MKN-1, and high levels of surface c-Met were found in MKN-28, MKN-45, SNU-5, and Hs746T.

c-Met CAR Jurkat cells were incubated with these GC cell lines to measure the IL-2 secreted by Jurkat cells. As expected, IL-2 levels were increased when c-Met CAR Jurkat cells were incubated with c-Met positive GC cells, except with Hs746T cells (Figure 3A). c-Met CAR Jurkat cells incubated with c-Met negative cell lines did not secrete IL-2 at all. In addition, IL-2 secretion by c-Met CAR Jurkat cells was blocked by purified soluble c-Met proteins (Figure 3B). These results indicate that our c-Met CAR construct specifically recognizes c-Met positive GC cells.

**Figure 4:**
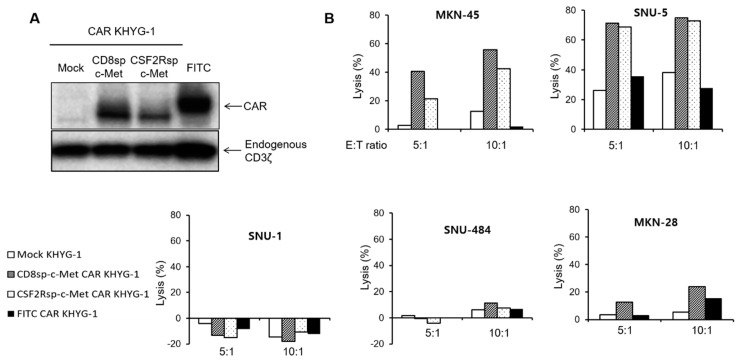
c-Met CAR KHYG-1 specifically lyses the c-Met positive GC cells. (**A**) Western blot with the cell lysates of mock CD8sp-c-Met, CSF2Rsp-c-Met, or FITC KHYG-1 cells to see the CAR expression (**B**) The mock CD8sp-c-Met CAR, CSF2Rsp-c-Met CAR, or FITC CAR KHYG-1 were co-incubated with MKN-45, SNU-5, SNU-1, and SNU-484 at E:T ratio of 5:1 or 10:1 for 5 h. The cytotoxicity of CAR KHYG-1 was measured by the Bright-Glo (luciferase) assay system. (The whole western blots figure see Figure S1).

We assessed the antigen-specific cytotoxicity of the anti-c-Met CAR KHYG-1 cell. The KHYG-1 cell is a NK/T cell line that has cytotoxic activity against target cells [28]. Because the KHYG-1 cell is easy to culture and easily transduced by lentiviral particles, we performed the cytotoxicity assay with the KHYG-1 cell before performing it with CAR T cells from primary T cells. The mock c-Met CAR or FITC CAR vectors were transduced into KHYG-1 cells using lentivirus. After 2 weeks of puromycin selection, the expression of each CAR in the KHYG-1 cells was confirmed by Western blot and FACS analysis (Figure 4A and Figure S2). The mock KHYG-1, c-Met CAR KHYG-1, or FITC CAR KHYG-1 cells were incubated with GC cells at the indicated E/T ratios. The c-Met CAR KHYG-1 showed a higher cytotoxic effect against c-Met positive GC cells (SNU-5, MKN-45, MKN28) than mock KHYG-1 or FITC CAR KHYG-1 (Figure 4B). c-Met CAR KHYG-1 cells did not eliminate the c-Met negative GC cell lines (SNU-1 and SNU-484). These data mean that our c-Met CAR constructs are qualified for developing CAR T cells against c-Met overexpressed GC cells.

### 2.3. The c-Met CAR T Cells Show Cytotoxicity Only against c-Met Positive GC Cells

**Figure 5:**
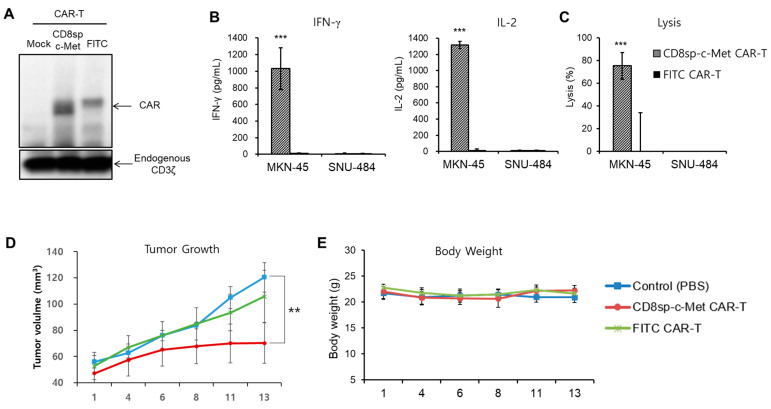
c-Met CAR T cells effectively kill MKN-45 both in vitro and in vivo. T cells were activated with CD3/CD28 Dynabeads and were cultured in medium containing rhIL-7 and rhIL-15. Two days post-activation, T cells were transduced with c-Met CAR or FITC CAR lentivirus. (**A**) At 9 days post-activation, cell lysates were prepared for Western blot to see CAR expression. (**B**) CD8sp-c-Met CAR or FITC CAR T (2 × 10^5^ cells) were incubated with MKN-45 or SNU-484 (2 × 10^4^ cells). The next day, supernatants were collected to perform IFN-γ and IL-2 ELISA. (**C**) CD8sp-c-Met CAR or FITC CAR T (1.2 × 10^5^ cells) were co-incubated with MKN-45 or SNU-484 (1.5 × 10^4^ cells) at E:T ratio 8:1 for 20 h. The bars show mean ± S.D. of experiments performed in triplicate. Statistical analysis was performed by the paired *t*-test. ** *p* ≤ 0.05; *** *p* ≤ 0.01, compared with the FITC CAR-T group. (**D**,**E**) After irradiating 3 Gy to NSG (NOD.Cg-PrkdcscidIl2rgtm1Wjl/SzJ) mice, MKN-45 cells were implanted subcutaneously into the right flank of mice. Nine days after tumor implantation, control (PBS, *n* = 5), CD8sp-c-Met CAR T (*n* = 5), or FITC CAR T (*n* = 5) cells were intratumorally administrated. (**D**) Tumor volume and (**E**) weight of mice were assessed at 2–3-day intervals. The bars show mean ± S.E. of the experiment performed in 5 mice. Statistical analysis was performed by one-way ANOVA followed by Dunnett’s multiple comparisons test. ** *p* ≤ 0.05; *** *p* ≤ 0.01, compared with the control (PBS) group. (The whole western blots figure see Figure S1).

Since our c-Met CAR construct was proved to be effective in Jurkat cells and KHYG-1 cells, we decided to produce c-Met CAR T cells with primary T cells. Peripheral blood mononuclear cells (PBMCs) were prepared from healthy donor’s blood, and lentiviral particles containing the CAR construct were infected to primary T cells by spinoculation. As shown in Figure 5A, both the c-Met CAR protein and FITC CAR protein were successfully expressed in T cells. To know if this c-Met CAR T cell is activated by the target cell, we measured the level of cytokine secretion by the CAR T cells incubated with the GC cell lines. c-Met CAR T cells secreted high levels of IFN-γ and IL-2 when incubated with MKN-45, a c-Met positive cell line; however, few cytokines were secreted when incubated with SNU-484, a c-Met negative cell line (Figure 5B). In addition, anti-c-Met CAR T cells lysed only c-Met positive gastric cancer cells, not c-Met negative gastric cancer cells (Figure 5C and Figure S3). This means that our c-Met CAR T cells function properly, so we decided to study the anti-tumor effect of c-Met CAR T cells in an in vivo mice model.

### 2.4. The c-Met CAR T Cells Suppress Tumor Growth of c-Met Positive GC In Vivo

To assess the anti-tumor effect of c-Met CAR T cells in vivo, we used the NSG (NOD.Cg-PrkdcscidIl2rgtm1Wjl/SzJ) mice model. NSG mice were irradiated with 3 Gy, and 1 × 10^7^ cells of MKN-45 cells were implanted subcutaneously in the right flank of the mice the next day. Nine days after tumor implantation, mice were randomly sorted into three groups (five mice per group) and PBS (control), 2 × 10^6^ cells c-Met CAR T, or FITC CAR T cells were intratumorally injected. Tumor size was measured at 2–3-day intervals (Figure 5D). Tumor growth was effectively inhibited only in mice injected with c-Met CAR T cells. On the other hand, mice injected with the control or FITC CAR T cells did not inhibit tumor growth. To evaluate the toxicity of CAR T cells, we measured the body weight of the mice (Figure 5E). There was no significant body weight change in mice administered the control (PBS) or the c-Met CAR T or FITC CAR T cells.

## 3. Discussion

CAR T cell therapies are attracting enormous attention because of their impressive effect on B-ALL patients [29]. Although the striking efficacy of CAR-based cell adoptive immunotherapy has been observed in hematological cancers, limited effect has been reported on solid tumors [30]. There are several reports on treating gastric cancer cells with CAR T cells. These CAR T cells targeted overexpressed antigens in gastric cancer, such as ICAM-1, PSCA, Her2, mesothelin, claudin18.2, folate receptor, and NKG2D ligand. [31,32,33,34,35,36,37] In this study, we noticed c-Met as a potent target for CAR T cells against GC, as c-Met is highly expressed in about 20% of GC patients [38]. c-Met CAR T cells have been created and evaluated for anti-cancer activity in breast cancer and liver cancer [39,40]. To dissect the availability of anti-c-Met CAR T cell for anti-cancer therapy in gastric tumors, we tested anti-c-Met CAR T cells on various gastric cancer cell lines. We created two anti-c-Met CAR constructs, CD8sp-c-Met CAR and CSF2Rsp-c-Met CAR. These c-Met CAR constructs have different signal peptides. In our experiment, both constructs showed similar effectiveness. In this paper, we used two cell lines, Jurkat and KHYG-1 cells. Due to the difficulties of making CAR T cells with primary T cells, we tested our constructs with the Jurkat and KHYG-1 cell lines before making CAR T cells from primary T cell. The Jurkat cell line is a CD4+ T cell line, which secretes IL-2 on activation, so many researchers have used this cell to study CAR T cells. KHYG-1 is a NK/T cell line which has the ability to lyse the target cells on activation. The KHYG-1 cell line is much easier to culture than the NK-92 NK cell line which is used widely among researchers [41]. Several research groups, including our group, reported that KHYG-1 cells exhibit excellent anti-cancer activity when CAR is incorporated [32,42,43]. The expression of c-Met in GC cell lines was measured, then cell lines were categorized as positive or negative based on the expression of c-Met.

We first tested CAR constructs using the IL-2 ELISA. IL-2 was secreted only when c-Met CAR Jurkat cells were co-cultured with c-Met positive GC cells. We were interested in whether the surface expression level of the target cell is correlated with the level of CAR T cell activation. Our data demonstrate that CAR T cells are never activated when incubated with the c-Met negative cell line. However, our data also demonstrate that the expression level of surface c-Met is not correlated with the level of CAR T cell activation. For example, although MKN-45 expresses more surface c-Met proteins than MKN-28, CAR Jurkat cells secrete more cytokine when incubated with MKN-28. It is a very interesting phenomenon, but currently we are not able explain why this happens. We guess that CAR T cells need something else other than the c-Met antigen to recognize target cells. Furthermore, although the Hs746T cell has a high level of c-Met, little IL-2 was secreted by the c-Met CAR Jurkat cells co-cultured with the Hs746T cell. Asaoka et al. reported that the Hs746T cell has a c-Met gene mutation causing juxtamembrane domain deletion [44]. We anticipate that this could be one reason why our c-Met CAR Jurkat cell was not effective on the Hs746T cell. We need to study further to know the exact reason why Hs746T cells were not responsive to c-Met CAR Jurkat.

The cytotoxicity of c-Met CAR KHYG-1 cells against the target cells was measured. c-Met CAR KHYG-1 cells effectively recognized only c-Met positive GC cells and showed cytotoxic effects. It is interesting that, although c-Met CAR Jurkat cells were activated more by MKN-45 than by SNU-5, c-Met CAR KHYG-1 cells lysed SNU-5 more than MKN-45. Moreover, although MKN-28 induced c-Met CAR Jurkat cells to secrete the maximal IL-2 level, MKN-28 was not eradicated as efficiently as MKN-45 and SNU-5. Currently, we have no idea why there is no correlation between IL-2 production and the lytic activity of CAR T cells in response to GC positive cell lines. We created another c-Met CAR cell with different scFvs called 5D5 (patent WO 2006/015371 A2). This scFv has a short linker sequence between a variable heavy chain (V_H_) and a variable light chain (V_L_). Figure S4 showed that c-Met CAR cells made by 5D5 exhibited low activity against gastric cancer cells both in ELISA and cytotoxic assay. Based upon these Jurkat and KHYG-1 cell data, we are assured that our c-Met CAR construct is qualified for developing CAR T cells from primary T cells. We created c-Met CAR T cells from primary T cells by lentivirus spinoculation. In the cytokine release assay, c-Met CAR T cells secreted large amounts of IL-2 and IFN-γ when incubated with c-Met positive GC cells. Almost no IL-2 was released by the CAR T cells when co-cultured with the c-Met negative cell line. These data mean that our c-Met CAR T cell selectively recognizes c-Met positive cancer cells. In the xenograft mouse model, the c-Met CAR T cells effectively suppressed the growth of MKN-45, a c-Met positive GC cell. In contrast, mice injected with PBS or FITC CAR T cells did not inhibit tumor growth. Although anti-Her2 CAR T cells have been injected intratumorally to breast cancer patients in clinical trials, intratumoral injection of CAR T cells is not a proper therapy for gastric cancer patients. In addition, our xenograft model does not recapitulate gastric cancer as well as the orthotopic model. Based on our in vitro and in vivo data, in this paper, we suggest that anti-c-Met CAR T cell therapy might work on gastric cancer cells. In further studies, we need to emphasize how to effectively apply anti-c-Met CAR T cells to gastric cancer patients.

Although c-Met is frequently overexpressed in various neoplastic cells, it is also known to be expressed in normal epithelial cells [45]. Therefore, it is a concern that c-Met CAR T cells may cause on-target off-tumor toxicity. Anti Her2 CAR T cell therapy has caused the death of a patient due to the low levels of Her2 in normal epithelial cells [46]. In addition, GD2 CAR T cells have caused fatal encephalitis in a neuroblastoma patient, as GD2 has low expression in the brain [47]. In further studies, to avoid these toxicities, we need to adapt dual CAR strategies to prevent CAR T cells from attacking normal tissues [48,49,50]. In this paper, although we found a potent target, c-Met, for CAR T cell therapy in gastric cancer, it is not easy to find success with CAR T cell therapy against solid tumors. As mentioned in the Introduction, many obstacles prevent CAR T cells from attacking solid tumors. In further research, we will examine how to make c-Met CAR T cells successful in solid tumors using genetic manipulation.

## 4. Materials and Methods

### 4.1. Cell Line

Jurkat and Hs746T cell lines were purchased from ATCC (Manassas, VA, USA). The KHYG-1 cell line was purchased from DSMZ (Braunschweig, Germany). MKN-1, MKN-45, SNU-1, SNU-5, and SNU-484 cell lines were purchased from KCLB (Seoul, Korea). The MKN28 cell line was purchased form JCRB (Tokyo, Japan). All the gastric cancer cells and Jurkat cells were maintained in RPMI 1640 medium (HyClone, Logan, UT, USA, #SH30027) supplemented with 10% fetal bovine serum (Gibco, Thermo Fisher Scientific, Dublin, Ireand, #16000-044). KHYG-1 cells were cultured in RPMI 1640 supplemented with 10% FBS and 200 IU/mL rhIL-2 (R&D Systems, Minneapolis, MN, USA, #202-IL-500). T cells were cultured in RPMI 1640 medium containing 10 ng/mL of IL-7 (PeproTech #200-07) and 10 ng/mL of IL-15 (PeproTech, Cranbury, NJ, USA, #200-15). All the cells were incubated in a humidified incubator at 37 °C with 5% CO_2_.

### 4.2. Construction of CAR Plasmid

The antigen-specific CAR constructs were based on the second-generation CAR composed of the scFv region, human CD28 transmembrane domain, human CD28 cytoplasmic domain, and human CD3ζ cytoplasmic domain. Each CAR construct was synthesized from Macrogen (Seoul, Korea).

CAR Jurkat cells were created by transient transfection of the CAR encoding vector, which is a pLVX-IRES ZsGreen vector (Clontech, Mountain View, CA, USA, #632187), into Jurkat cells. The CAR KHYG-1 cell line was made by lentiviral particles containing c-Met CAR or FITC CAR encoding the pLVX-CMV-Puro vector (Clontech #632164). The pLVX-CMV-puro plasmid was digested with XhoI/EcoRI or XhoI/BamHI restriction enzymes.

CAR T cells were created by lentiviral particles containing pLVX-EF1α vector. c-Met CAR or FITC CAR genes were incorporated into the pLVX-CMV-IRES-ZsGreen1 (Clontech #632187) vector. The ClaI/XbaI (c-Met CAR) or XhoI/BamHI (FITC CAR) restriction enzymes were used to replace the CMV promoter with an EF1α promoter. Also, XbaI/MluI (c-Met CAR) or BamHI/MluI (FITC CAR) sites were cut to delete the IRES-ZsGreen1 region of the pLVX-EF1α-IRES-ZsGreen1 vector to minimize vector size.

Primers used for the polymerase chain reaction (PCR) were as follows. CD8sp-c-Met CAR forward primer: 5′ ATC TAG CTC GAG ATG GCC CTG CCC GTG ACC GCC CTG CTG CTG CCC CTG GCC CTG CTG CTG CAC GCC GCC CGG CCC CAG GTG CAG CTG GTG CAG TC 3′, CSF2Rsp-c-Met CAR forward primer: 5′ ATC TAG CTC GAG ATG CTG CTG CTG GTG ACC TCC CTG CTG CTG TGC GAG CTG CCC CAC CCC GCC TTC CTG CTG ATC CAG GTG CAG CTG GTG CAG TC 3′, c-Met CAR reverse primer: 5′ ATC TAG GAA TTC TTA GCG AGG GGG CAG GGC CT 3′, FITC CAR forward primer: 5′ ATC TGC CTC GAG ATG CTG CTG CTG GTG ACC TC 3′, and FITC CAR reverse primer: 5′ AAT ATT GGA TCC TTA GCG AGG GGG CAG GGC 3′.

### 4.3. Lentivirus Production and Generation of CAR KHYG-1

To generate the lentiviruses, c-Met CAR and FITC CAR vectors were co-transfected with lentiviral packaging plasmids, pRSV-Rev (Addgene, Watertown, MA, USA, #12253), pMDLg/pRRE (Addgene #12251), and pMD2.G (Addgene #12259) to 293 T cells. The supernatants containing lentivirus were collected and filtered using a 0.45 μm PES membrane filter (Millex-HP #SLHP033RB). The lentivirus supernatants were concentrated using Lenti-X concentrator (Clontech #631232), mixed with 8 μg/mL of polybrene in KHYG-1 cells, and centrifuged at 1000× *g*, for 90 min. For selection of transduced cells, KHYG-1 cells were treated with puromycin at 1 μg/mL for several weeks.

### 4.4. Human T Cell Isolation and Generation of CAR T Cell

Whole blood was obtained from Korean Red Cross Blood Services and peripheral blood mononuclear cells (PBMCs) were isolated using density gradient centrifugation. Whole blood was diluted with an equal volume of PBS containing 2% FBS. Then, 15 mL of Lymphoprep (STEMCELL Technologies, Vancouver, BC, Canada, #07851) was added to the conical centrifuge tube, followed by the addition of 30 mL of diluted whole blood. Blood samples were centrifuged at 800× *g* for 20 min continuously without brake and only the yellow middle layer (mononuclear cells, MNCs) was transferred to a new conical centrifuge tube. After adding the wash buffer (PBS with 2% FBS) to the MNCs, it was centrifuged for 8 min at 300× *g* (repeated twice).

To activate the T cells in vitro, T cells were cultured in RPMI1640 medium with Dynabeads Human T-Activator CD3/CD28 (Thermo Fisher Scientific #11132D) and rhIL-2. After 2 days, the Dynabeads were removed using an EasySep Magnet (STEMCELL Technologies #18001). T cells were mixed with c-Met CAR or FITC CAR lentivirus supernatants, respectively. Then, 8 μg/mL of polybrene was added to the T cell-lentivirus mixture and spinoculation was conducted for 90 min at 1000× *g*. After centrifugation, the supernatant was removed and the transduced T cells were incubated in a medium containing rhIL-7 and rhIL-15.

### 4.5. Cytotoxicity Assay

The cytotoxic activities of the CAR KHYG-1 cells were measured using the Bright-Glo Luciferase Assay System (Promega, Madison, WI, USA, #E2650). The luciferase gene was transfected into target GC cells. The next day, mock KHYG-1, c-Met CAR KHYG-1, or FITC CAR KHYG-1 cells were co-cultured with GC cells expressing luciferase at an E:T ratio of 5:1 or 10:1. After 5 h of co-culture, the luciferase Bright-Glo Luciferase assay reagent was added to the effector/target cell mixture. Finally, plates were shaken for 5 min at room temperature, then luminescence was measured via an EnVision reader (PerkinElmer, Watertown, MA, USA). Cytotoxic activity was calculated as Formula (1):

(1) $$\left(1-\frac{\mathrm{Luminescence}\left[\mathrm{Target}\;\mathrm{cells}\;\mathrm{with}\;\mathrm{Effector}\;\mathrm{cells}\right]}{\mathrm{Luminescence}\left[\mathrm{Target}\;\mathrm{cells}\;\mathrm{only}\right]}\right)\times 100$$

### 4.6. Cytokine Release Assay

To measure cytokine secretion, 1.5 × 10^5^ mock Jurkat, c-Met CAR Jurkat cells, FITC CAR Jurkat, c-Met CAR T cells, or FITC CAR T cells were co-cultured with 1 × 10^4^ GC cells in 96-well plates (E:T ratio of 15:1). After overnight incubation at 37 °C, only the supernatants were collected. The amounts of human interleukin-2 (IL-2) and human interferon gamma (IFN-γ) were measured using a human IL-2 and IFN-γ (BioLegend, San Diego, CA, USA, #431801 and #430104) ELISA kit.

### 4.7. Evaluation of Surface c-Met Protein Level

Approximately 1 × 10^6^ cells were washed with cold PBS and resuspended in FACS resuspension buffer I (PBS, 10% FBS, 0.02% sodium azide). Anti-human HGF R/c-MET antibody (R&D Systems #MAB3582) was added to the cells and the cells were incubated on ice for 30 min. After washing with cold PBS, FACS resuspension buffer II (PBS, 3% bovine serum albumin) containing a secondary antibody was added to the cells and incubated for 30 min on ice. The cells were washed with cold PBS and resuspended in FACS resuspension buffer III (PBS, 3% BSA, 0.02% sodium azide) supplemented Hoechst 33342 (Thermo Fisher Scientific #H3570). After incubation at 37 °C for 15 min, c-Met expression was analyzed using the NucleoCounter NC-3000 (ChemoMetec, Allerød, Denmark).

### 4.8. Western Blotting

To confirm expression of the CAR construct and c-Met protein, the cell lysates of mock Jurkat, c-Met CAR Jurkat, mock KHYG-1, c-Met CAR KHYG-1, FITC CAR KHYG-1, c-Met CAR T, and FITC CAR T were harvested with a 1X SDS-PAGE loading buffer (10% glycerol, 2% SDS, 50 mM Tris-HCl (pH 6.8), 3% β-mercaptoethanol). Lysates were boiled for 10 min at 95 °C and were loaded into 4–15% gradient Mini-PROTEAN TGX gels (BioRad, Hercules, CA, USA, #456-1086). CAR expression was detected by CD3ζ (BD Biosciences, Franklin Lakes, NJ, USA, #551034). GAPDH (Cell Signaling Technology, Danvers, MA, USA, #5174) bands were used as loading control.

### 4.9. FACS Analysis

The mock CD8sp-c-Met CAR or FITC CAR KHYG-1 (1 × 10^6^ cells) were suspended in 100 μL of cold stain buffer (PBS 0.2% BSA, 0.08% sodium azide) and stained with 5 μL of Fc-tagging human HGF R/c-MET protein (AcroBiosystems, Newark, DE, USA, #MET-H5256) for 20 min on ice. After washing with the cold buffer, 5 μL of PE anti-human IgG Fc (BioLegend #409304) was added to the cells and incubated for 20 min on ice. The cells were rinsed with the cold buffer and fixed using 4% formaldehyde solution for 15 min at 4 °C. After washing and filtering the cells, FACS analysis was done with a BD FACSCanto II (BD Biosciences).

### 4.10. Mouse Xenograft Model

All of the procedures were conducted according to guidelines approved by the Laboratory Animal Care and Use Committee of the Korea Research Institute of Chemical Technology. The NSG (NOD.Cg-PrkdcscidIl2rgtm1Wjl/SzJ) mice used in this experiment were purchased from Charles River Laboratories, Inc. (Singapore) After total body irradiation (3 Gy), MKN-45 GC cells (1 × 10^7^ cells/mouse) were injected subcutaneously in the right flank of the mice the next day. When the volume of a tumor reached about 50 mm^3^, control (PBS), c-Met CAR T and FITC CAR T cells (2 × 10^6^ cells/mouse) were administered intratumorally. After CAR T cell administration, tumor volume was measured at intervals of 2 to 3 days using a caliper, and the volume was calculated using the following formula: volume = length × width^2^ × 0.5. The body weight of the mice was measured using animal scales (and balance) at intervals of 2 to 3 days.

## 5. Conclusions

In summary, our c-Met CAR T cells effectively inhibit c-Met positive GC cells in vivo as well as in vitro. Not only gastric cancer, but also other cancers, such as prostate, colon, and breast, have a high expression of c-Met [19,20,21,22,23]. Therefore, c-Met CAR T cells could play a key role in anti-cancer therapy in solid tumors.

## Data Availability

The data presented in this study are available on request from the corresponding author.

## Supplementary Materials

The following are available online at https://www.mdpi.com/article/10.3390/cancers13225738/s1, Supplementary material S1: DNA sequences of CAR segments, Figure S1: The whole western blots figures, Figure S2: To confirm the surface expression of c-Met CAR in KHYG-1 cells, cells were stained with Fc-tagging human c-Met protein followed by incubation with PE anti-human IgG Fc, Figure S3: Cytotoxic assay was performed with c-Met positive GC cell lines using c-Met CAR T cells, Figure S4: The anti-tumor activity of another c-Met CAR T cells made by 5D5 scfv.

## Abbreviations

ALL	Acute lymphoblastic leukemia
CAR	Chimeric antigen receptor
GC	Gastric Cancer
scFv	Single chain variable fragment
IFN-γ	Interferon-gamma
IL-2	Interleukin-2
FITC	Fluorescein isothiocyanate
ELISA	Enzyme-linked immunosorbent assay
NK	Natural killer
NSG	NOD.Cg-PrkdcscidIl2rgtm1Wjl/SzJ
PBMC	Peripheral blood mononuclear cell
