# Supplementary material for: c-Met-Specific Chimeric Antigen Receptor T Cells Demonstrate Anti-Tumor Effect in c-Met Positive Gastric Cancer
Source: Cancers (Basel). 2021 Nov 16;13(22):5738. doi: 10.3390/cancers13225738 (PMC8616279; doi:10.3390/cancers13225738)
Supplement: Supplementary file 1 [file cancers-13-05738-s001.zip › Supplementary Material S1 and Figures S2-S4.pdf]

cancers

Supplementary Materials

c-Met-Specific Chimeric Antigen Receptor T Cells Demonstrate Anti-Tumor Effect in c-Met Positive Gastric Cancer

Chung Hyo Kang, Yeongrin Kim, Da Yeon Lee, Sang Un Choi, Heung Kyoung Lee and Chi Hoon Park

Supplementary Material S1: DNA sequences of CAR segments

c-Met scfv

caggtgcagctggtgcagtctggagcagaggtgaaaaagcccggggagtctctgag-
gatctcctgtcagggttctggatacagttttcccacccactggatcacctgggtgcgccagatgcccgggaaaggcctggagtggatgggaacgattgatcctactgactctta
caatttctatggaccgtcgttccaaggccacgtcaccatctcagccgacagctccagcag-
caccgcctacctgcagtggagcagcctgaaggcctcggacaccgccatgtattactgtgcgagagatggcaactactatgatagtcgcggttattactacgatacttttgatat
gtggggccaagggacactggtcaccgtctcctcaggactaggaggactaggaggaggag-
gaagtggaggaggaggaagtggaggaagtagtggagtgggatccgacatccagatgacccagtctccatccttcctctctgcatctgtcggagacagagtcaccatcactt
gccgggccagtcagggcatcagtacttatttagcctggtatcaacaaaaac-
cagggacagcccctaaactcctgatctattctgcatccactttggaaagtggggtcccatcgcgattcagcggaagtggatccgggacagatttcactctcaccatcagcagcc
tgcagcctgaagattctgcaacttactattgtcaacaggctgacagtttcccgctcac-
tttcggcggagggaccaaggtggagatcaaacgtggaggagccagcctcgtggaa

CD28

gcggccgcaattgaagttatgtatcctcctccttacctagacaatgagaagagcaatggaac-
cattatccatgtgaaagggaaacacctttgtccaagtcccctatttcccggaccttctaagcccttttgggtgctggtggtggttgggggagtcctggcttgctatagcttgctagt
aacagtggcctttattattttctgggtgaggagtaagaggagcaggctcctgcacagtgac-
tacatgaacatgactccccgccgccccgggcccacccgcaagcattaccagccctatgccccaccacgcgacttcgcagcctatcgctcc

CD3

agagtgaagttcagcaggagcgcagacgcccccgcgtaccagcagggccagaaccagctc-
tataacgagctcaatctaggacgaagagaggagtacgatgttttggacaagagacgtggccgggaccctgagatggggggaaagccgagaaggaagaaccctcagga
aggcctgtacaatgaactgcagaaagataagatggcggaggcctacagtgagattgg-
gatgaaaggcgagcgccggaggggcaaggggcacgatggcctttaccagggtctcagtacagccaccaaggacacctacgacgcccttcacatgcaggccctgccccctc
gc

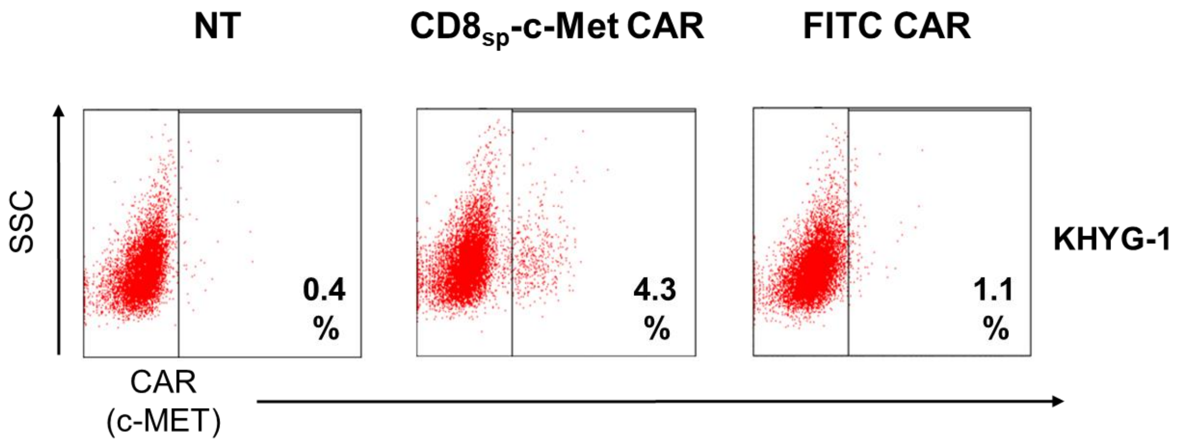

Figure S2. To confirm the surface expression of c-Met CAR in KHYG-1 cells, cells were stained with Fc-tagging human c-Met protein followed by incubation with PE anti-human IgG Fc.

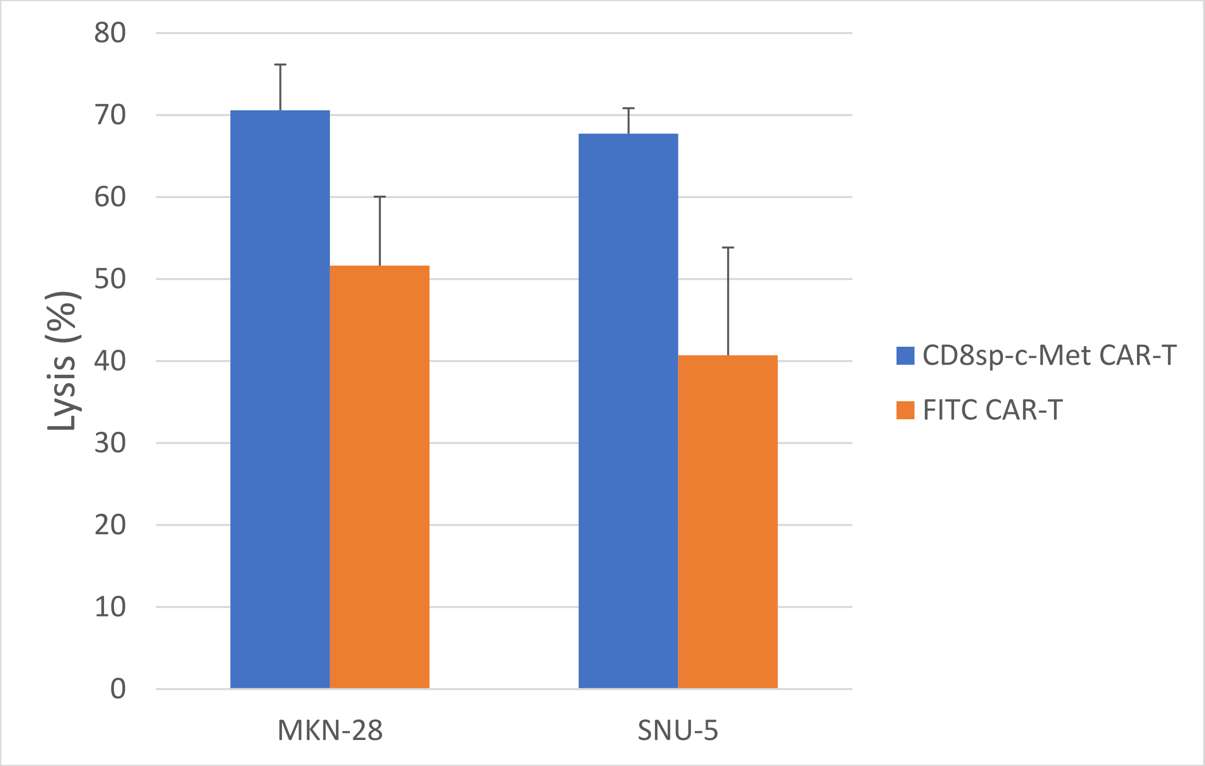

Figure S3. Cytotoxic assay was performed with c-Met positive GC cell lines using c-Met CAR T cells.

A.

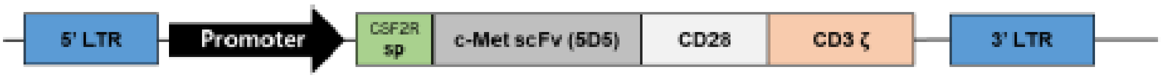

B. 5D5 sequence

GACATCCAGATGACCCAGTCCCCCTCCTCCCTGTCCGCCTCCGTGGGCGACCGGGTGACCATCACCTGCAAGT
CCTCCCAGTCCCTGCTGTACACCTCCTCCCAGAAGAACTACCTGGCCTGGTACCAGCAGAAGCCCGGCAAGG
CCCCCAAGCTGCTGATCTACTGGGCCTCCACCCGGGAGTCCGGCGTGCCCTCCCGGTTCTCCGGCTCCGGCTC
CGGCACCGACTTCACCCTGACCATCTCCTCCCTGCAGCCCGAGGACTTCGCCACCTACTACTGCCAGCAGTAC
TACGCCTACCCCTGGACCTTCGGCCAGGGCACCAAGGTGGAGATCAAGCGGGGCGGCGGCTCCGAGGTGCA
GCTGGTGGAGTCCGGCGGCGGCCTGGTGCAGCCCGGCGGCTCCCTGCGGCTGTCCTGCGCCGCCTCCGGCTA
CACCTTCACCTCCTACTGGCTGCACTGGGTGCGGCAGGCCCCCGGCAAGGGCCTGGAGTGGGTGGGCATGAT
CGACCCCTCCAACTCCGACACCCGGTTCAACCCCAACTTCAAGGACCGGTTCACCATCTCCGCCGACACCTC
CAAGAACACCGCCTACCTGCAGATGAACTCCCTGCGGGCCGAGGACACCGCCGTGTACTACTGCGCCCGGTA
CGGCTCCTACGTGTCCCCCCTGGACTACTGGGGCCAGGGCACCCTGGTGACCGTGTCCTCC

C.

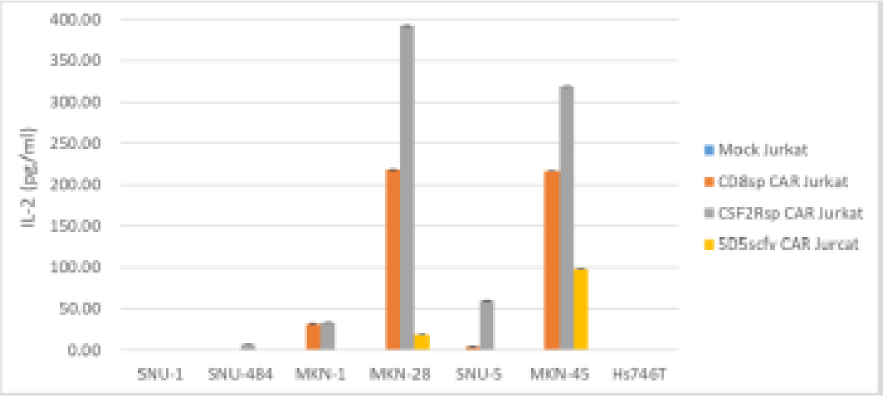

D.

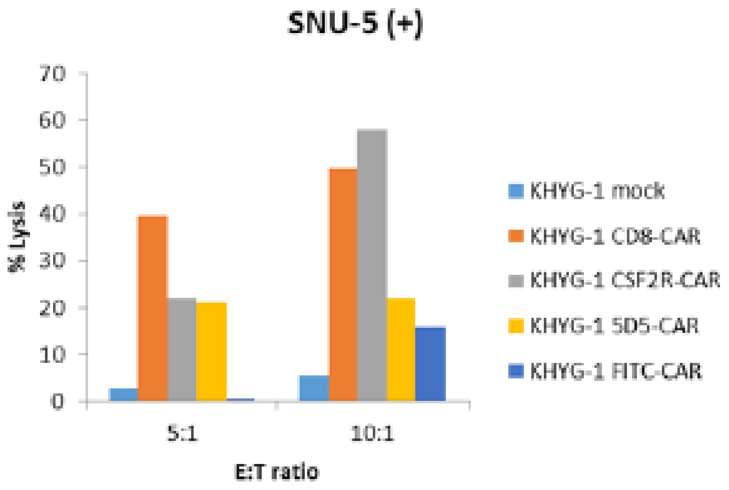

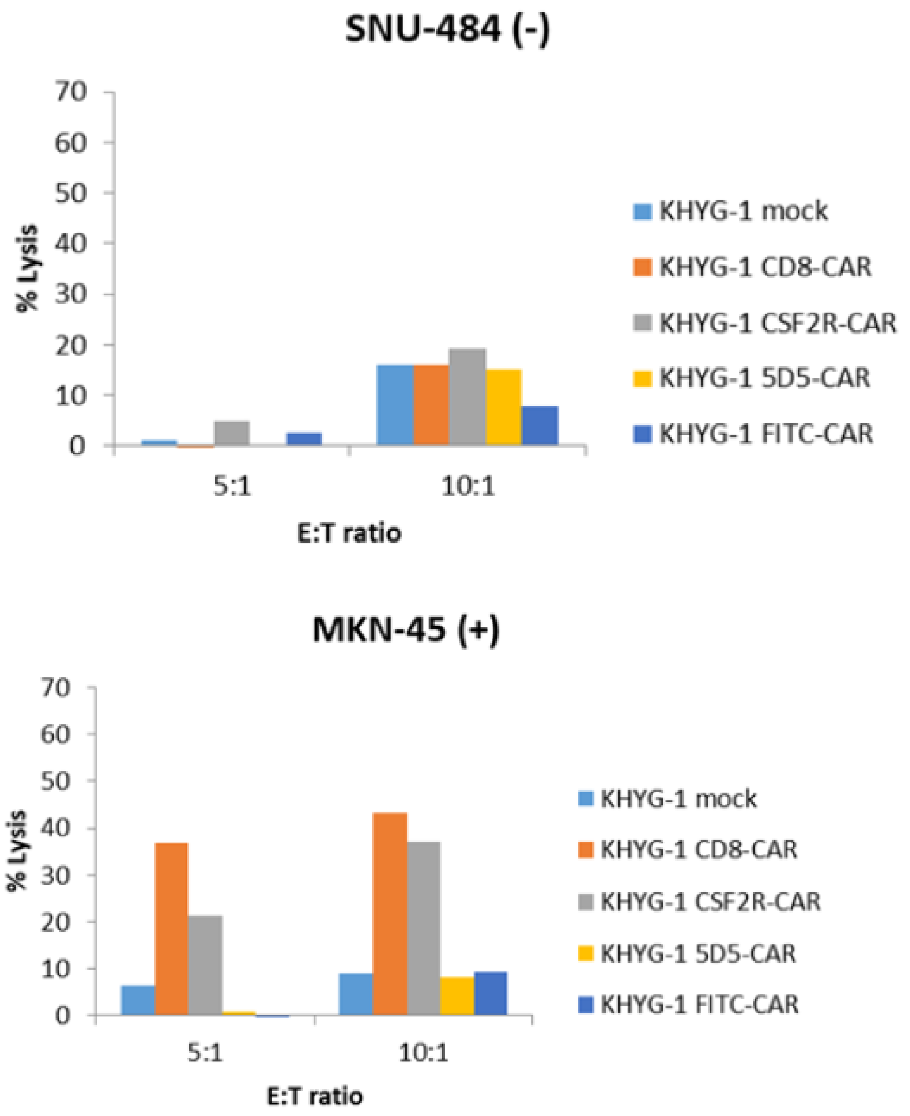

Figure S4. The anti-tumor activity of another c-Met CAR T cells made by 5D5 scfv.

Copyright: © 2021 by the authors. Licensee MDPI, Basel, Switzerland. This article is an open access article distributed under the terms and conditions of the Creative Commons Attribution (CC BY) license (http://creativecommons.org/licenses/by/4.0/).
