# Supplementary figures and images for: c-Met-Specific Chimeric Antigen Receptor T Cells Demonstrate Anti-Tumor Effect in c-Met Positive Gastric Cancer
Source: Cancers (Basel). 2021 Nov 16;13(22):5738. doi: 10.3390/cancers13225738 (PMC8616279; doi:10.3390/cancers13225738)

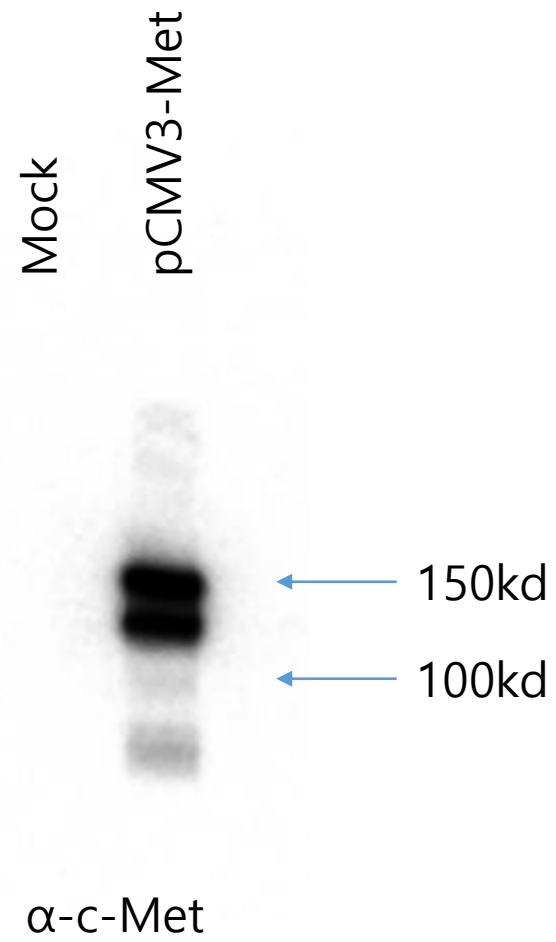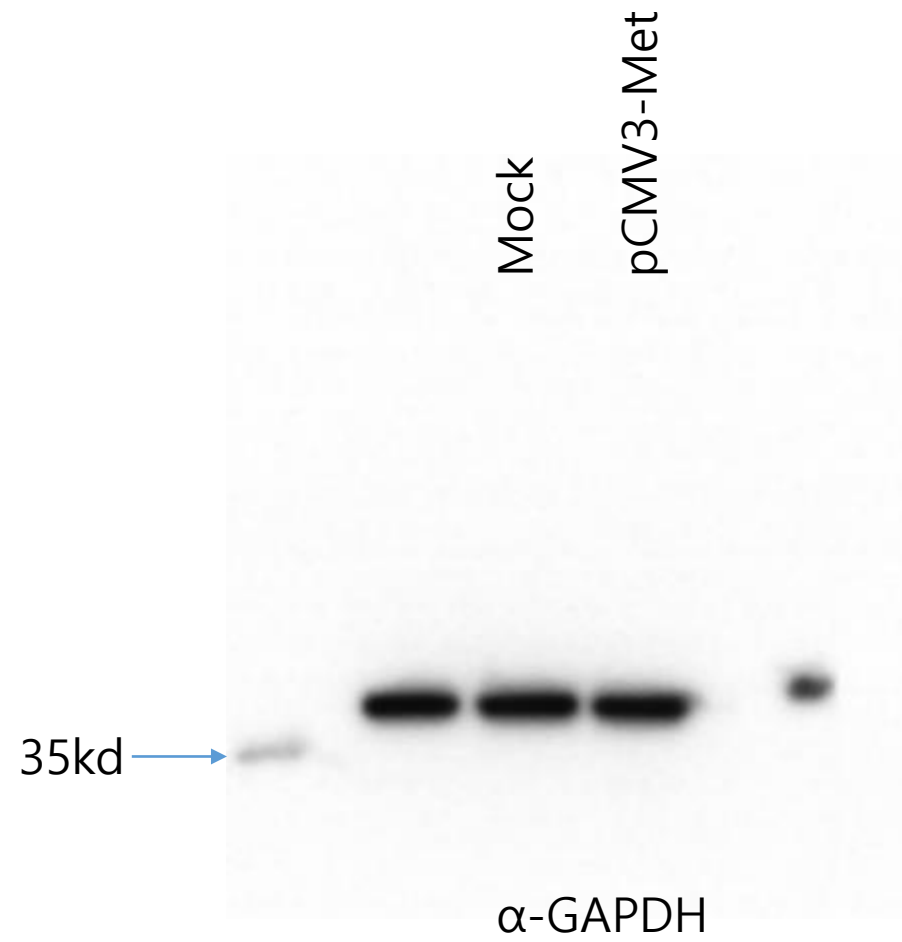

Figure 1

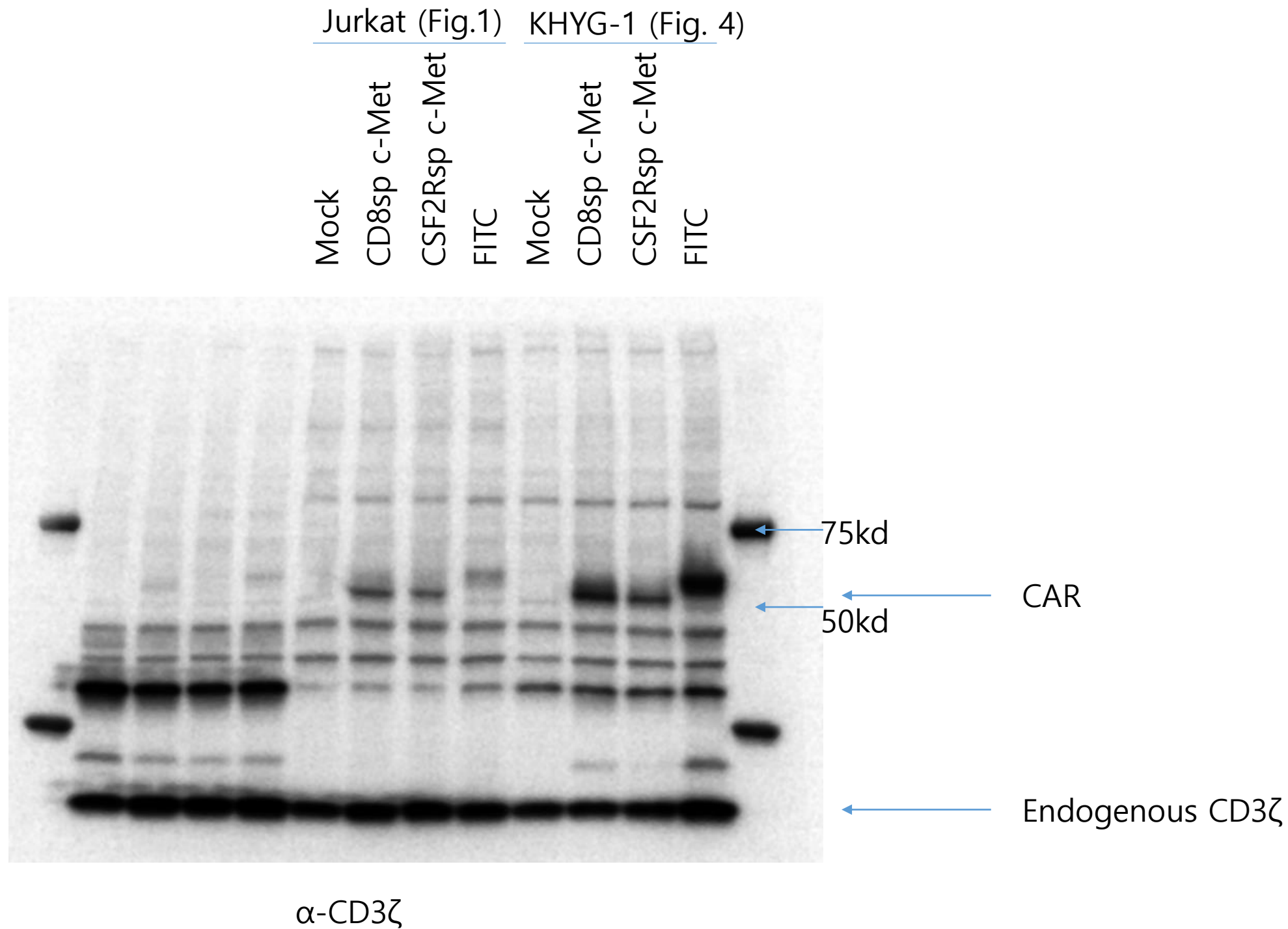

Figure 1, 4

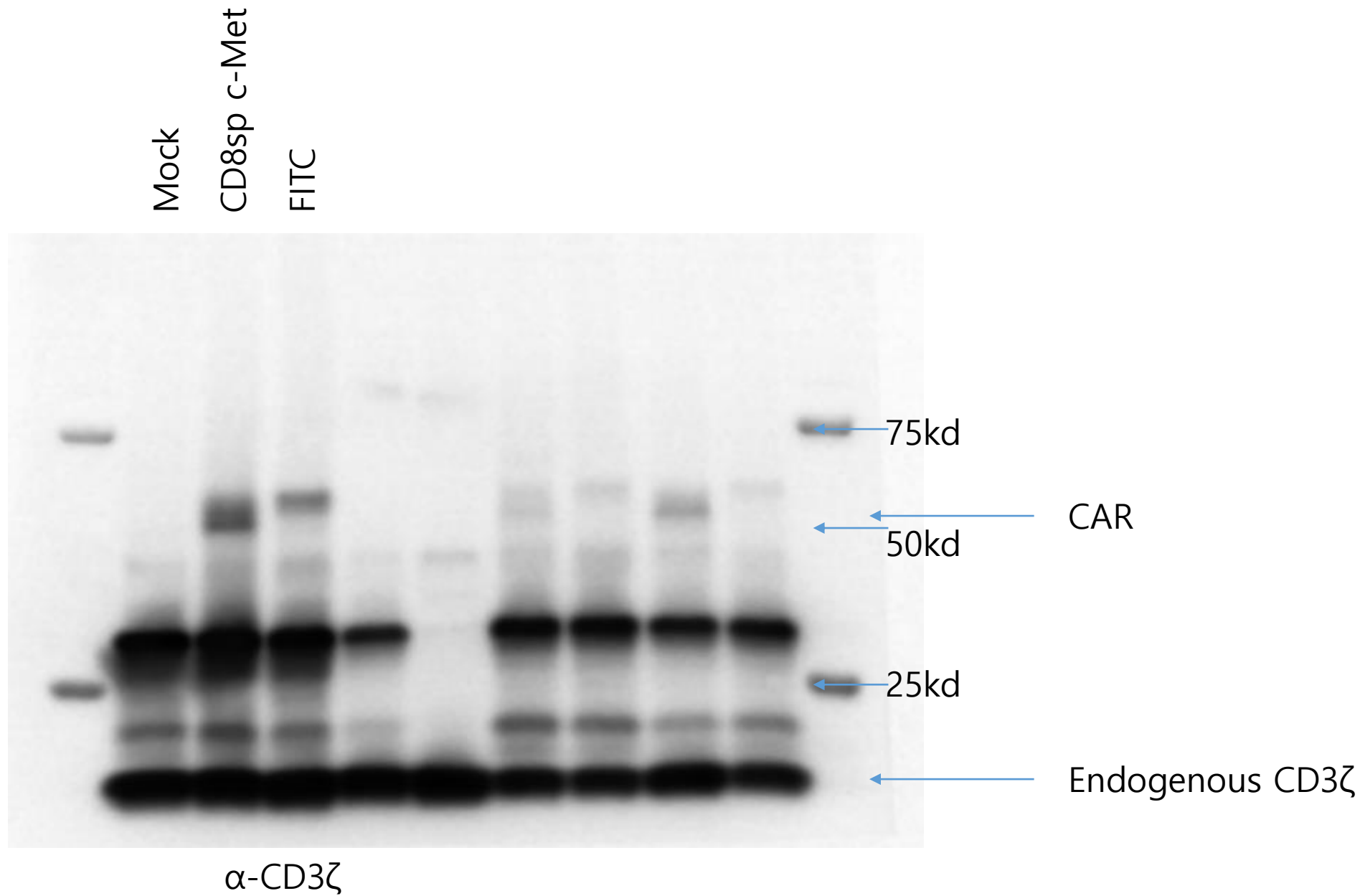

Figure 5

Supplement: Supplementary file 1 [file cancers-13-05738-s001.zip › Figure S1.pdf]
